# A simple model for behaviour change in epidemics

**DOI:** 10.1186/1471-2458-11-S1-S3

**Published:** 2011-02-25

**Authors:** Fred Brauer

**Affiliations:** 1 Department of Mathematics, University of British Columbia, Vancouver, BC, V6T 1Z2, Canada

## Abstract

**Background:**

People change their behaviour during an epidemic. Infectious members of a population may reduce the number of contacts they make with other people because of the physical effects of their illness and possibly because of public health announcements asking them to do so in order to decrease the number of new infections, while susceptible members of the population may reduce the number of contacts they make in order to try to avoid becoming infected.

**Methods:**

We consider a simple epidemic model in which susceptible and infectious members respond to a disease outbreak by reducing contacts by different fractions and analyze the effect of such contact reductions on the size of the epidemic. We assume constant fractional reductions, without attempting to consider the way in which susceptible members might respond to information about the epidemic.

**Results:**

We are able to derive upper and lower bounds for the final size of an epidemic, both for simple and staged progression models.

**Conclusions:**

The responses of uninfected and infected individuals in a disease outbreak are different, and this difference affects estimates of epidemic size.

## Introduction

During the course of an epidemic, there are changes in behaviour which have an effect on the transmission of infection. Individuals who are infected may make fewer contacts with others because the debilitating effects of their illness or because of advice by public health organizations to stay home in order to avoid infecting others. Individuals who have not been infected may take hygienic measures to reduce the risk of being infected and may take other steps such as avoidance of large public gatherings. There is evidence that such measures had substantial effects during the 1918 influenza pandemic [1].

The question of what factors influence people to change their behaviour is a difficult one, probably more in the areas of psychology and sociology than epidemiology and public health. In this study, we avoid this question, and assume only reduction of contacts sufficient to transmit infection by members of the population. Since the factors affecting such behaviour changes are different for those who are infected and those who wish to avoid becoming infected, it is necessary to assume different fractional reductions in these two groups. This implies that, even in a model in which mixing is assumed homogeneous without behavioural change, it is necessary to recognize that the mixing becomes heterogeneous, and this may affect the behaviour of the model.

In this note, our purpose is to estimate the effect that given reductions in contacts have on the final size of an epidemic, without trying to model the factors that might cause such reductions. It would be more realistic to assume that the rate or amount of behavioural change, at least for uninfected members of the population, is dependent on some information about the extent of the epidemic, perhaps the number of infectious people or the total number of reported disease deaths. Study of such questions is one of the most important gaps in scientific knowledge of the spread of communicable diseases. One contribution in the direction of studying this area is [4]

## A simple *SIR* epidemic model

The simplest special case of the Kermack-McKendrick epidemic model is(1)

in which it is assumed that contact between individuals satisfies a mass action law with members making a constant number *βN* contacts in unit time, that there is an exponential distribution of infected periods with mean length 1/*α*, and that there are no disease deaths (so that the total population size *N* remains constant) [5]. We assume that initially the population consists of *S*_0_ susceptible members and a (presumably small) number *I*_0_ of infectious members, with *S*_0_ + *I*_0_ = *N*. For the model (1) it is known that the basic reproduction number is

that the number of susceptibles decreases to a positive limit *S_∞_* and the number of infectious members decreases to zero as *t → ∞*, and that the attack rate, the extent of the epidemic which is defined as

satisfies the final size relation(2)

This was originally derived in [5], although the basic reproduction number was not given explicitly. More recent derivations may be found in [2,6].

Since (1) is a two-dimensional autonomous system of differential equations, the natural approach would be to find equilibria and linearize about each equilibrium to determine its stability. However, since every point with *I* = 0 is an equilibrium, the system (1) has a line of equilibria and this approach is not applicable (the linearization matrix at each equilibrium has a zero eigenvalue). It is possible to analyze the system in the phase plane (the (*S*, *I*) plane) obtaining a phase portrait and also the final size relation. Although this derivation of the final size relation is simple and has a useful geometric interpretation, it does not generalize readily to more complicated compartmental models. For this reason, we give also an analytic argument which does generalize.

Because *S* is a decreasing non-negative function, it has a limit *S_∞_* ≥ 0 as *t* → ∞*.* The sum of the two equations of (1) is

(*S + I*)′ *=* –*αI.*

Thus *S + I* is a non-negative smooth decreasing funtion and therefore tends to a limit as *t → ∞*. Also, it is not difficult to prove that the derivative of a smooth decreasing function must tend to zero, and this shows that

Thus *S + I* has limit *S_∞_*.

Integration of the sum of the two equations of (1) from 0 to *∞* gives

Division of the first equation of (1) by *S* and integration from 0 to *∞* gives the final size relation (2). We now modify the model (1) by assuming that susceptible members decrease their rate of contact by a fraction *p*, 0 *≤ p ≤* 1 and that infectious members decrease their rate of contact by a fraction *q*, 0 *≤ q ≤* 1. As different subgroups of the population now have different activity levels, we must specify the mixing between groups. Since the population is assumed to mix homogeneously in the absence of disease, we assume proportionate mixing. Thus we assume that the number of contacts in unit time made by susceptible members, infectious members, and removed members are, respectively,

*pβN*, *qβN*, *βN*,

and the fraction of contacts made by susceptible members that are with infectious members is

It is convenient to define

*T = pS + qI + R*,

so that the rate of new infections is

and the model is given by the pair of differential equations(3)

From the fact that (*S + I*)*′ =* –*αI* it follows that *I →* 0 as *t → ∞*, and from integration of this equation it follows that(4)

Then integration of the equation for *S* in (3)(5)

From min(*p*, *q*)*N ≤ T ≤ N* it follows that

and this, combined with (4), and (5), gives(6)

It is easy to see that

and this, together with (6), gives a final size inequality(7)

For the model (3), the next generation matrix calculation [7] shows that

while the reproduction number if there were no behavioural change would be

so that

*R*_0_ = *qR^∗^ ≤ R^∗^*.

We define *R*_1_, *R*_2_ by

Then (7) takes the form(8)

It is clear that

*R*_1_*≤ R*_0_, *R*_1_*≤ R^∗^*, *R*_0_*≤ R*_2_*≤ R^∗^,*.

In addition,

*R*_0_*< R^∗^* (*q <* 1), *R*_1_*< R^∗^* (*pq <* 1), *R*_1_*< R*_0_ (*p <* 1)

*R*_2_*< R^∗^* (*p <* 1, *q <* 1), *R*_2_ = *R*_0_ (*p ≤ q*)*.*

## The final size equation

A final size equation of the form(9)

determines *S_∞_* as a function *S_∞_*(*R*) of *R*. It is easy to show [2] that the final size relation (9) has a unique solution *S_∞_* with

Using implicit differentiation of (9) and this estimate, it is easy to see that the function *S_∞_*(*R*) is strictly decreasing. The final size inequalities (8) imply that for the model (3) the final size *S_∞_* satisfies the inequalities

*S_∞_*(*R*_2_) *< S_∞_ < S_∞_*(*R*_1_)*.*

The behavioural response in the model (3) decreases the final susceptible population size from *S_∞_*(*R^∗^*) to *S_∞_*(*R*_2_) or less. If one ignores the heterogeneity in the model, one might assume that the final susceptible population size is *S_∞_*(*R*_0_). If *p > q*, so that *R*_2_*> R*_0_, it is possible that the final susceptible population size could be smaller than this. However, simulations suggest that the final susceptible population size is usually larger than *S_∞_*(*R*_0_).

For example we simulate the model (3) with parameters

*βN* = 0*.*45, *α* = 0*.*25, *p* = 0*.*9, *q* = 0*.*8,

so that

*R*_0_ = 1*.*44, *R^∗^* = 1*.*8, *R*_1_ = 1*.*30, *R*_2_ = 1*.*62*.*

A simulation gives *S_∞_* = 483*.*3. The reproduction number corresponding to this value of *S_∞_*, which we may call the effective reproduction number, denoted by *R_E_*, is 1*.*405. Further simulations suggest that the effective reproduction number is likely to be close to and somewhat smaller than *R*_0_. In fact, since *R*_2_ = *R*_0_ if *p ≤ q*, (7) may be replaced by(10)

Even if *p >q* the upper bound in (10) may be valid. Simulations suggest that the upper bound is valid except possibly when *p* is very close to 1, *q* is very close to zero, and *R*_0_ is well below 1. It is possible to find examples for which *R_E_ > R*_0_, such as *p* = 0*.*9, *q* = 0*.*2, which gives

*R*_0_ = 0*.*36, *R*_1_ = 0*.*324, *R*_2_ = 1*.*62, *R_E_* = 0*.*375*.*

This indicates that behavioural response of the type assumed here usually reduces the size of the epidemic a little more than might be expected by a naive approach.

## Staged progression epidemic models

It is not feasible to extend the results of the previous section to general age of infection epidemic models, but we can analyze the special case of staged progression models. We consider a *SI*_1_*I*_2_ ⋯ *I_n_* model in which the relative infectivity in stage *j* is *ε_j_*, and the rate of transfer to the next stage is *α_j_*. This leads to the model(11)

It is known [3] that, for this model,

We now add to the model (11) the assumption that susceptibles reduce contacts by a factor p, infectious members in stage *j* reduce their contacts by a factor *q_j_*, and that the mixing between groups is proportionate. Then the rate of contacts in unit time by susceptibles is *pβN*, and the fraction of these contacts that are with infectious members in stage *j* is *q_j_/T*, where

The resulting model is(12)

Then the next generation approach [7] shows that the basic reproduction number is

The reproduction number for the model (11) without behavioural response is

Integration of the equations for *I*_1_, *I*_2_, ⋯, *I_n_* in (12) gives(13)

while integration of the equation for *S* gives(14)

Also, integration of the sum of the equations for *S* and *I*_1_ in (12) gives(15)

Since *T*(*s*) *≤ N*, combination of (13), (14), (15) gives

with

To obtain an upper bound, we use the inequality

*T ≥* min(*p*, *q*_1_, *q*_2_, ⋯ , *q_n_*)*N.*

If min(*p*, *q*_1_, *q*_2_, ⋯ , *q_n_*) = *p*, then *p/T ≤*1*/N*, and we have

If min(*p*, *q*_1_, *q*_2_, ⋯ , *q_n_*) = *q_k_*, we obtain

with

We may summarize these calculations by saying that the bounds obtained for the simple *SIR* model extend to the staged progression model (12). There is no difficulty in extending to staged progression models with arbitrarily distributed length of stay in each stage. The mean time in stage *j* replaces 1*/α_j_* in each estimate. We have not carried out the calculations here because of the technical complications in writing the model equations, but these may be found in [3].

## Conclusions

Behavioural changes are an essential aspect of the course of an epidemic. The changes in behaviour by infectious members of a population have different causes than the changes in behaviour by uninfected members, and a model incorporating behavioural changes should reflect this. One implication is that a model incorporating behavioural changes must include heterogeneous mixing. One consequence of this is that the final size of an epidemic can not be determined exactly from a final size relation but can only be approximated. There is an effective reproduction number which is less than the basic reproduction number in many cases but not necessarily always.

Epidemic models with age structure or other heterogeneities in mixing can also be extended to incorporate behavioural changes. This would result in models with complicated mixing behaviour that would be difficult to analyze. There would be a system of final size equations which could not be solved exactly, but would still yield final size estimates. An important question that has not yet been attacked is the formulation of models that include behavioural responses, especially by uninfected members of the population, that depend on the state and history of the epidemic.

## Competing interests

The author declares that he has no competing interests.
